# Mechanical effects of taper angles in implant–abutment connection: a finite element study

**DOI:** 10.1186/s40729-025-00660-4

**Published:** 2026-01-16

**Authors:** Miho Tokumoto, Tatsuya Matsuzaki, Nobuo Sakai, Ikiru Atsuta, Yasunori Ayukawa

**Affiliations:** 1 Division of Oral Rehabilitation, Faculty of Dental Science, Kyushu University, 3-1-1 Maidashi, Higash-ku, Fukuoka, 812-8582 Japan; 2 Mechanical and Control Engineering, Kyushu Institute of Technology, 1-1 Sensui-machi, Tobata-ku, Kitakyushu, Fukuoka, 804-8550 Japan

**Keywords:** Dental implant, Taper angle, Implant–abutment connection, Finite element analysis, Press-fit, Abutment axial displacement, Preload loss, Microgap, Stress distribution

## Abstract

**Purpose:**

To analyze how the taper angle (defined here as the half‑angle per side) influences the mechanics of the implant–abutment connection using finite element analysis.

**Methods:**

Three‑dimensional finite element models (implant body, abutment, and screw) with taper angles of 8°, 15°, 18°, and 22° were established in ABAQUS/CAE. All components were modeled as linearly elastic Ti‑6Al‑4 V (E = 110 GPa, ν = 0.35). Frictional contact (μ = 0.3) was assigned at the taper and screw interfaces. A bolt load of 605 N (equivalent to 35 Ncm) was applied and then released to assess the press‑fit retention. Under the maintained preload, 100-N vertical and horizontal loads (unidirectional and bidirectional) were applied for five cycles. The primary outcomes are the abutment axial displacement, implant von Mises stress, bolt load change, and microgap size.

**Results:**

Smaller taper angles (8°, 15°) retained press‑fit after preload release, whereas larger angles (18°, 22°) lost press‑fit contact. As the taper angle decreased, the abutment axial displacement, implant stresses, and bolt load loss increased. The effect of the loading direction follows the order: vertical < horizontal (unidirectional) < horizontal (bidirectional). Microgaps decreased with smaller taper angles and cycling.

**Conclusion:**

Taper angle differences affect press-fit, abutment axial displacement, screw loosening, stress distribution, and microgap formation.

## Background

A taper joint implant system is a connection type with a conical interface between the implant body and the abutment joint [1]. Compared with other implant systems, taper joints are more resistant to preload loss (loosening) because screw tightening increases the interface contact and frictional wedging [2–5]. They also tend to exhibit smaller microgaps, potentially limiting bacterial ingress and peri‑implant bone loss [6–9]. However, a key concern with taper joints is abutment axial displacement, which occurs when screws are tightened or loads are applied [10–13]. Such displacement is expected to expand the implant body and cause excessive stress.

Taper angle differences are a key factor influencing abutment axial displacement, screw loosening, microgap formation, and excessive stress on the implant. The taper angle differs among implant manufacturers; however, comparative mechanical evaluations across angles remain limited, and angle-specific prosthetic protocols have yet to be established.

This study used the finite element method (FEM) to determine how the taper angle (per side) influences abutment axial displacement, screw loosening, stress distribution, and microgaps under clinically relevant loading.

In addition, this study systematically compares multiple taper angles that span both the press-fit and non–press-fit regimes, under clinically relevant cyclic loading conditions. This apprllows us to quantify the transitional mechanical behavior around thmit angle, providing novel insight into how taper geometry governs the shift from stae frictional engagement to loss of fit—an aspect that has not been clarified in previous FEM research.

## Methods

### Finite element modeling

This study used the finite element analysis software ABAQUS/CAE (Dassault Systèmes, Vélizy‑Villacoublay, France) for model design and analysis.

**Fig. 1:**
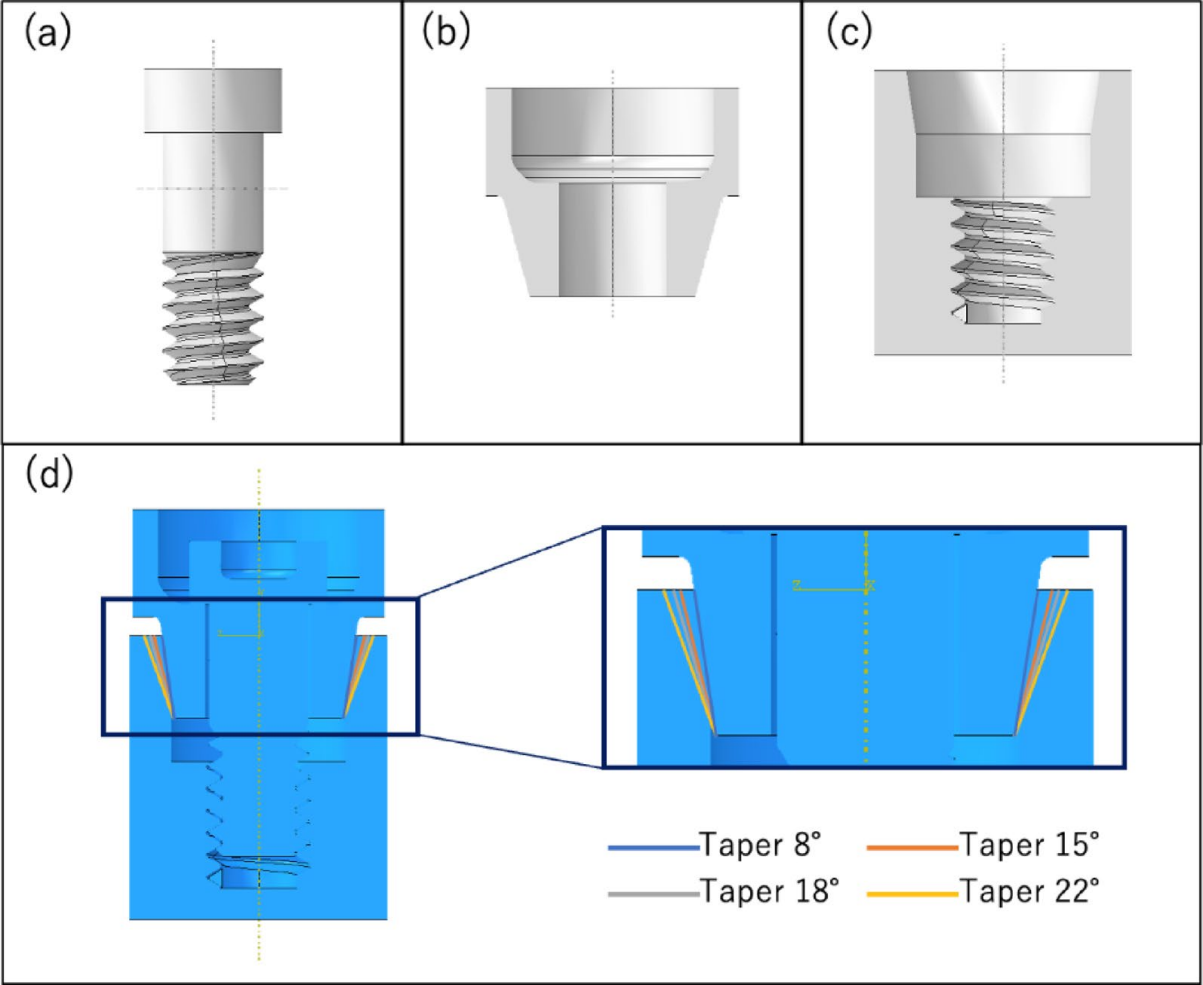
Examples of implant models: **a** abutment screw; **b** abutment; **c** implant body; **d** implant–abutment–screw complex and enlarged figure of joint. Color-coded by each taper angle

Three-dimensional models of implant components (implant body, abutment, and abutment screw) with different taper angles (8°, 15°, 18°, and 22°) were constructed. These component models were combined to create a composite implant model (Fig. 1).

The shape of the screw pitch is based on ISO 262,724, 68-1. Metal friction was assumed between the parts of the implant components (abutment–abutment screw, abutment–implant body, implant body–abutment screw), and the friction coefficient was set to 0.3. All components were assumed to be homogeneous, isotropic, and linearly elastic, composed of Ti‑6Al‑4 V (Young’s modulus of 110, Poisson’s ratio of 0.35) [14]. The mesh of the analytical parts was divided into elements using 10-node tetrahedral quadratic elements.

### Implant–abutment mechanics under bolt loading

The bolt load corresponding to a tightening torque of 35 Ncm, assuming a friction coefficient of 0.3, was calculated as follows [15] The tightening torque T is given by:

1 $$ T = \frac{{F_{f} \left\{ {\frac{{d_{2} }}{2}\left( {\frac{\mu }{{{\mathrm{cos}}\alpha }} + {\mathrm{tan}}\beta } \right) + \frac{{d_{n} }}{2}\frac{{\mu_{n} }}{{{\mathrm{cos}}\gamma }}} \right\}}}{1000} $$

The parameters were set as follows:

screw pitch, $$\:P=0.35\:\mathrm{m}\mathrm{m}$$;

$$ \begin{aligned} {\mathrm{screw}}\;{\mathrm{fundamental}}\;{\mathrm{triangle}}\;{\mathrm{height}},\;H  \\  = {{\sqrt 3 } \mathord{\left/ {\vphantom {{\sqrt 3 } 2}} \right. \kern-\nulldelimiterspace} 2}P = 0.303109\;{\mathrm{mm}} \quad \quad \quad \quad \quad \\ \end{aligned} $$

$$ {\text{screw diameter}},d = 1.6{\text{ mm}} $$

2 $$ {\text{effective diameter}},d_{2} = d - \left( {{\raise0.7ex\hbox{$H$} \!\mathord{\left/ {\vphantom {H 2}}\right.\kern-0pt} \!\lower0.7ex\hbox{$2$}} - {\raise0.7ex\hbox{$H$} \!\mathord{\left/ {\vphantom {H 8}}\right.\kern-0pt} \!\lower0.7ex\hbox{$8$}}} \right) = 1.37267{\text{ mm}} $$

3 $$ {\text{friction coefficient}},\mu = \mu_{n} = 0.3 $$

4 $$ {\mathrm{Half}} - {\text{angle of screw thread }}\alpha = 30^\circ $$

5 $$ {\text{lead angle}},\beta = {\raise0.7ex\hbox{$P$} \!\mathord{\left/ {\vphantom {P {\left( {\pi \times d_{2} } \right)}}}\right.\kern-0pt} \!\lower0.7ex\hbox{${\left( {\pi \times d_{2} } \right)}$}} = 0.081162 \,{\mathrm{rad}} $$

6 $$ {\text{screw seat effective diameter}},d_{n} = {\raise0.7ex\hbox{${\left( {1.6 + 2.2} \right)}$} \!\mathord{\left/ {\vphantom {{\left( {1.6 + 2.2} \right)} 2}}\right.\kern-0pt} \!\lower0.7ex\hbox{$2$}} = 1.9 \,{\mathrm{mm}} $$

screw seat angle,

7 $$\:\gamma\:=0\:\mathrm{r}\mathrm{a}\mathrm{d}=0\:^\circ\:$$

By substituting parameters (2–7) into Eq. (1), the tightening torque of $T=0.350041\text{ Nm}$ corresponds to a bolt load of ${F}_{f}=605 \,\mathrm{N}$.

**Fig. 2:**
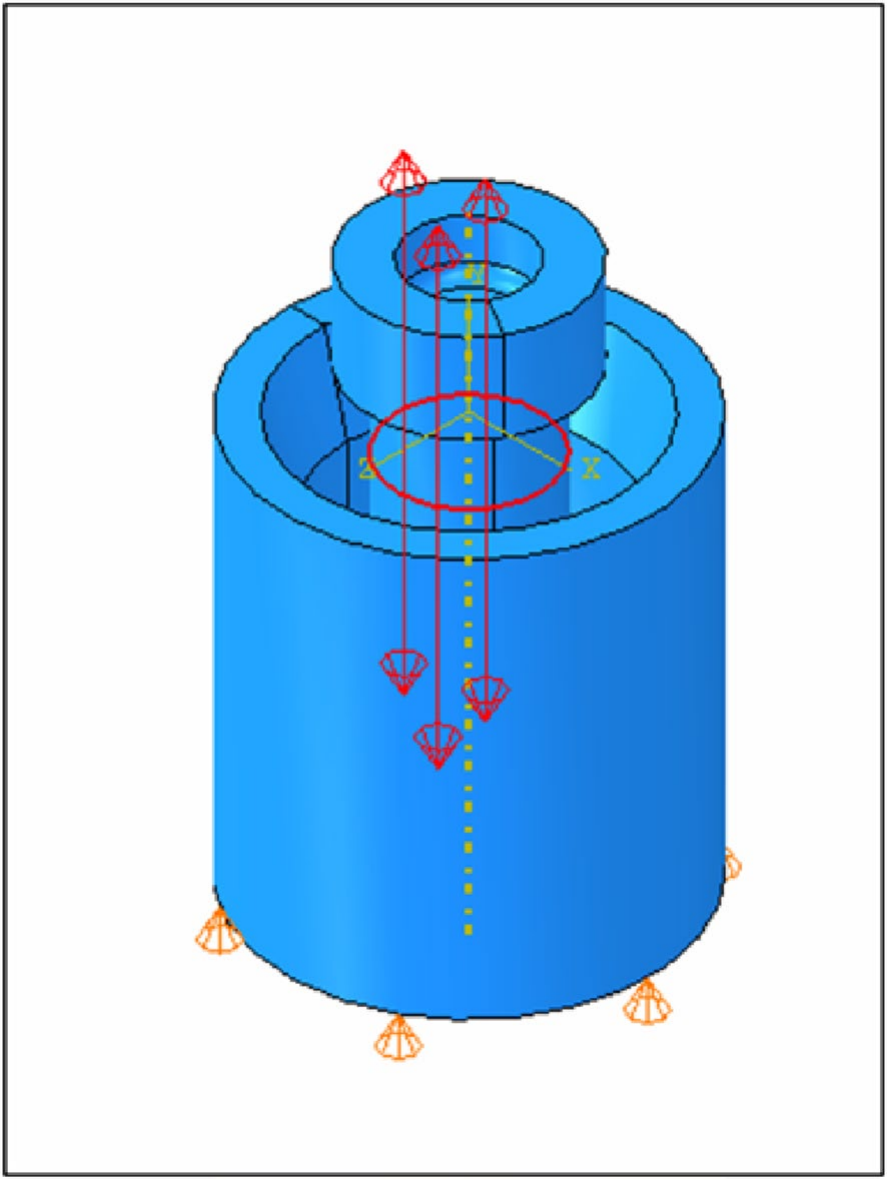
Constraint and bolt loading point

As the constraint conditions, the bottom of the implant body was fixed vertically, one central point was completely fixed, and one lateral point was fixed with anti-rotational fixation. The bolt load was loaded at a point with a distance of 0.9 mm from the abutment screw seating surface (Fig. 2).

The following bolt loading protocol was used: a bolt load of 605 N was ramped over 1.0 s (Step 1), held for 0.5 s (Step 2), released to 0 N over 1.0 s (Step 3), and held for 0.5 s (Step 4). The axial abutment axial displacement in Steps 1–4 was analyzed and plotted for each taper angle. Additionally, the stress distribution diagrams after Steps 2 and 3 were analyzed for each taper angle to check whether the abutment was fitted into the implant body by friction.

### Loading conditions

**Fig. 3:**
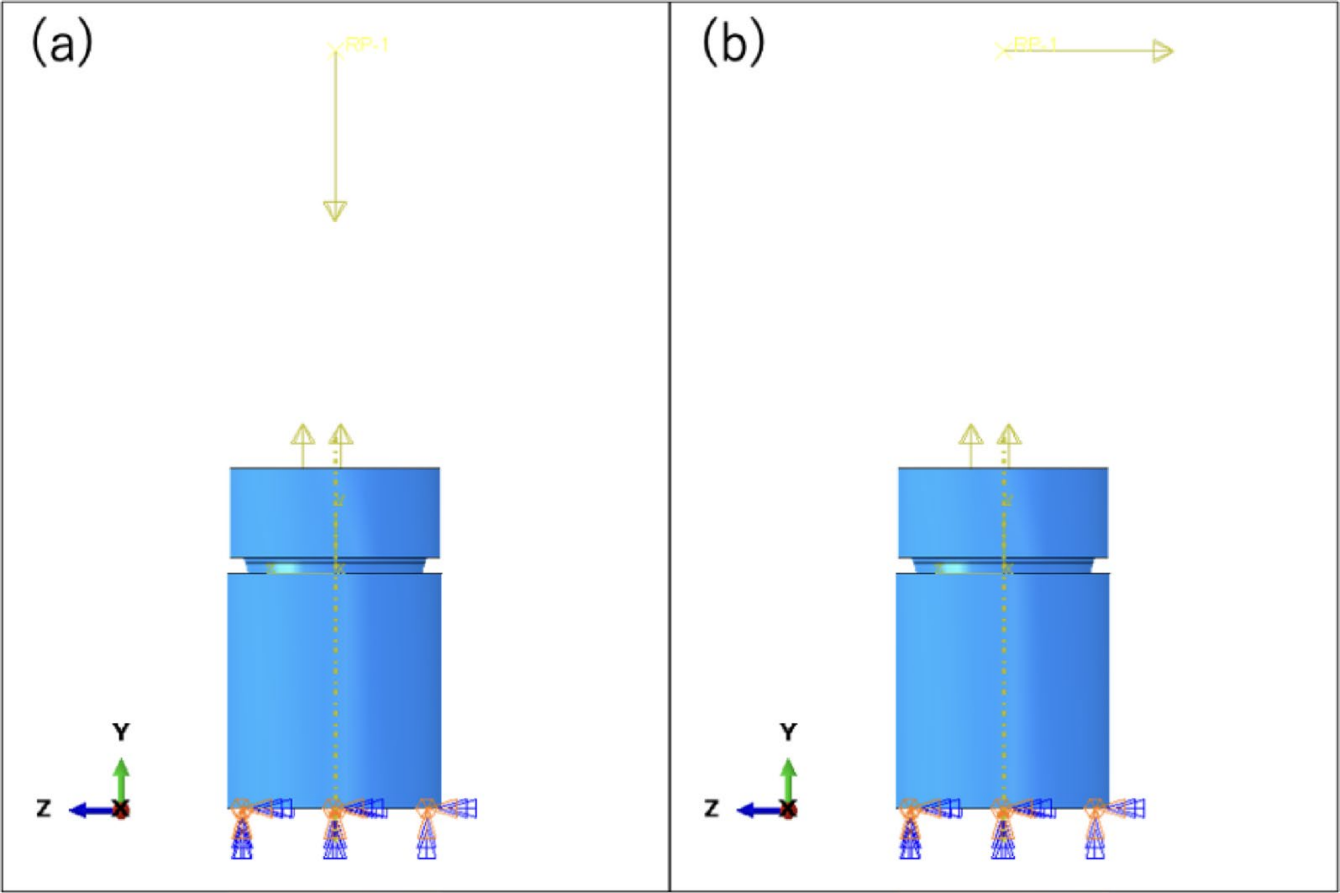
Constraint and loading point: **a** vertical load; **b** horizontal load

As the constraint conditions, the implant body was completely fixed while preserving the deformation of the bolt load at the bottom. External forces were applied to a remote reference point kinematically coupled to the abutment top surface (kinematic coupling) and positioned 10 mm above the implant platform. The global axes were defined with + Y along the implant axis; compressive vertical loads acted along − Y and horizontal loads along ± Z (Fig. 3).

Cyclic loading was initiated after the bolt load reached a stable state following the 0.5-s hold period. In each case, a peak force of 100 N was applied at the remote point and repeated for five cycles (Table 1).

 The vertical loads simulated the tapping movements, the unidirectional horizontal loads simulated the thrusting of the anterior teeth, and the bidirectional horizontal loads simulated the lateral movements of the molars.

**Table 1:** The vertical loads simulated the tapping movements, the unidirectional horizontal loads simulated the thrusting of the anterior teeth, and the bidirectional horizontal loads simulated the lateral movements of the molars

Case	Magnitude	Direction (Axis)	Application height	Time profile per cycle	Cycles
Vertical	100 N	− Y	10 mm	1.0 s − Y on → 1.0 s off	5
Horizontal (unidirectional)	100 N	− Z	10 mm	1.0 s − Z on → 1.0 s off	5
Horizontal (bidirectional)	100 N	± Z	10 mm	0.5 s − Z on → 0.5 s off → 0.5 s + Z on → 0.5 s off	5

### Implant–abutment mechanics under repeated loading

Loading was applied according to the conditions described in the previous subsection, and the axial abutment displacement and bolt load changes over time were analyzed and plotted for each angle.

### Microgap

**Fig. 4:**
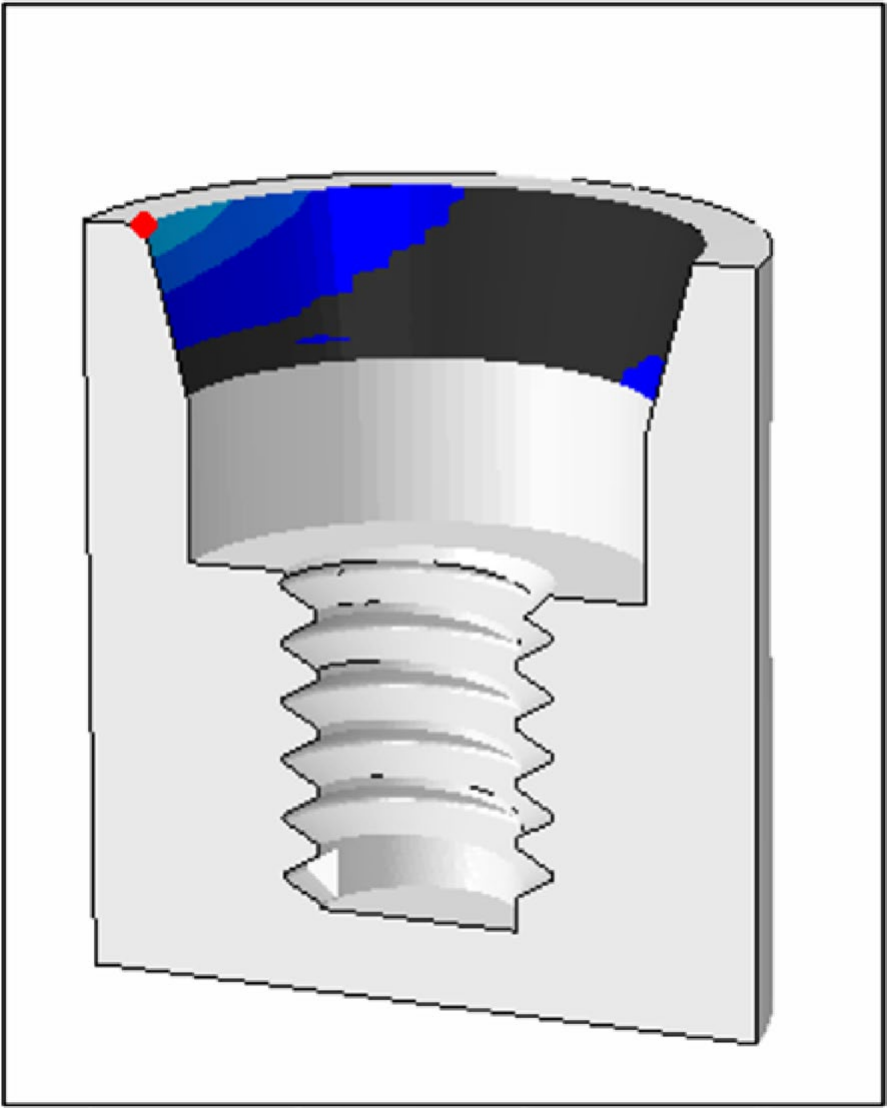
Microgap measurement point

The points at which the microgap was most separated were measured during the first horizontal load, fifth unidirectional horizontal load, and fifth bidirectional horizontal load (Fig. 4).

The amount of separation and contact between the implant body and the abutment was visualized and compared for each angle.

## Results

### Implant–abutment mechanics under bolt loading

**Fig. 5:**
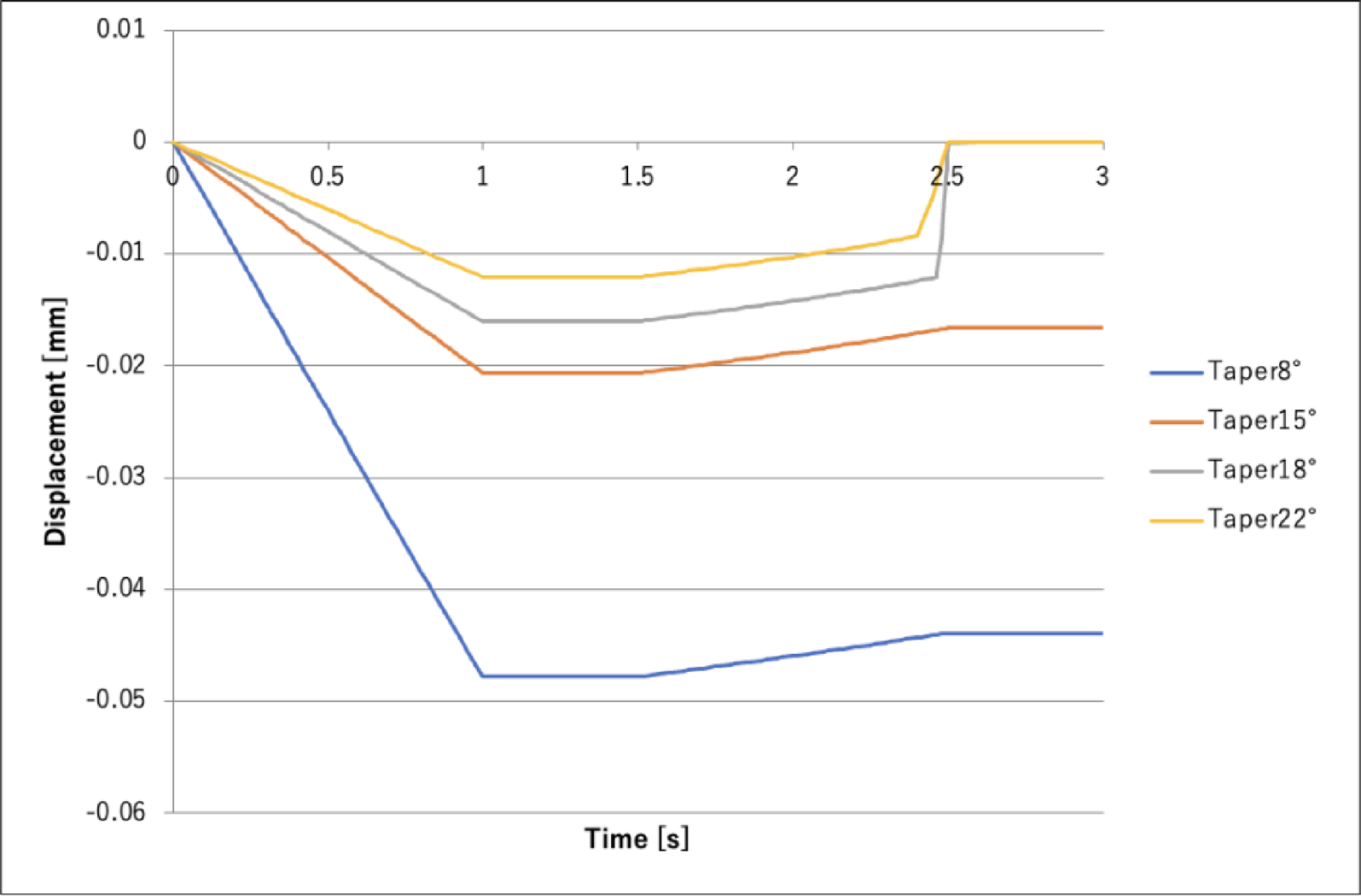
Axial abutment displacement before and after bolt load application

Figure 5 shows a plot of abutment displacement over time.

**Fig. 6:**
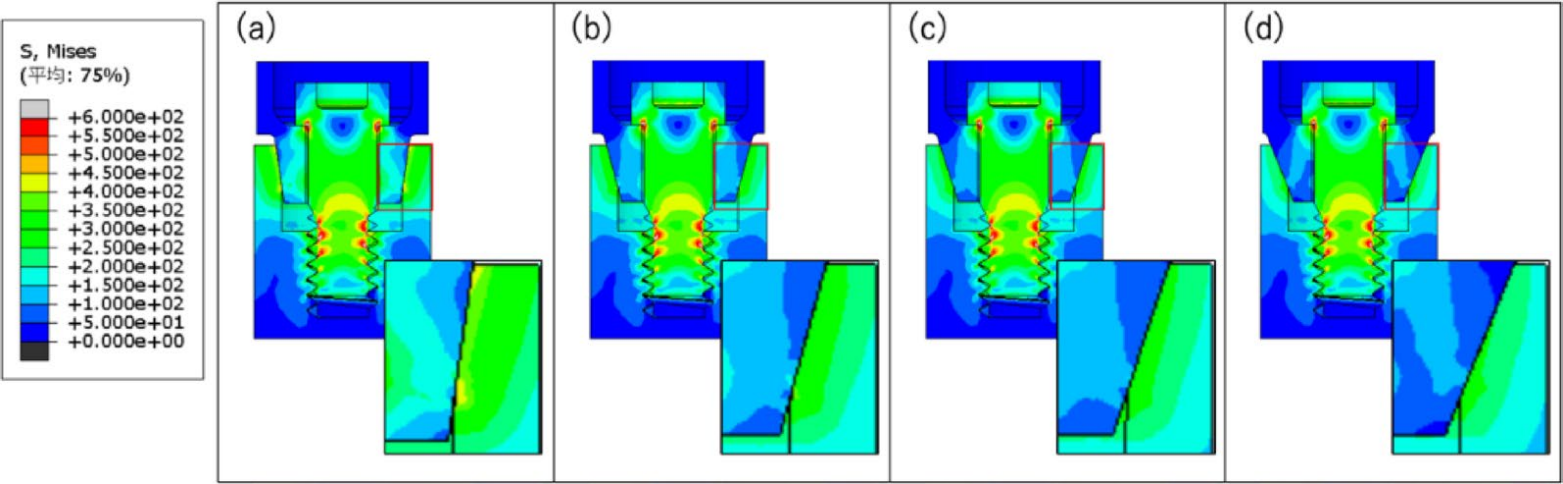
Diagram of stress distribution after bolt load application: **a** taper 8°; **b** taper 15°; **c** taper 18°; **d** taper 22°

**Fig. 7:**
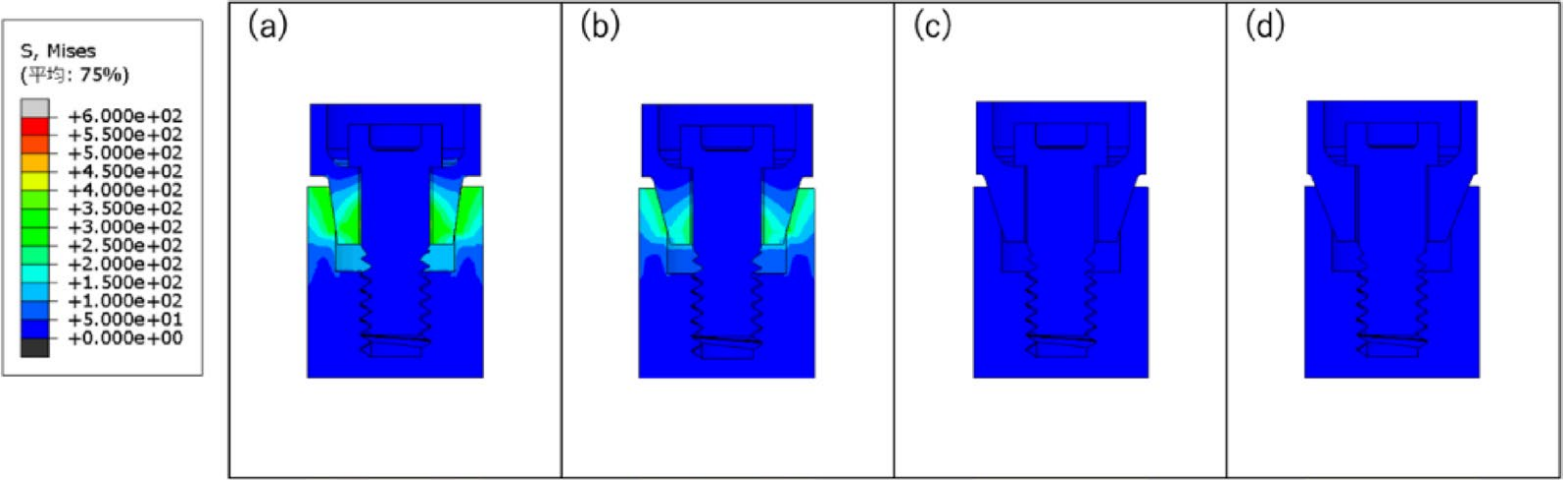
Diagram of stress distribution after bolt load removal: **a** taper 8°; **b** taper 15°; **c** taper 18°; **d** taper 22°

The stress distribution diagram at the end of Step 2 is shown in Fig. 6, and the stress distribution diagram at the end of Step 3 is shown in Fig. 7.

Figure 5 shows that, as the taper angle becomes smaller, the amount of abutment axial displacement and rate of increase in displacement become greater. Figure 6 shows that the stress on the implant body increases as the taper angle becomes smaller. In Fig. 7, with taper angles of 8° and 15°, the contact pressure remains even after the bolt load is released, suggesting that the implant body and abutment are fitted together by friction. Additionally, the contact pressure at 8° was greater than that at 15°, indicating that the abutment was more firmly fitted into the implant body at 8°. In contrast, with taper angles of 18° and 22°, contact pressure was not generated, indicating abutment detachment from the implant body.

### Implant–abutment mechanics under repeated loading

**Fig. 8:**
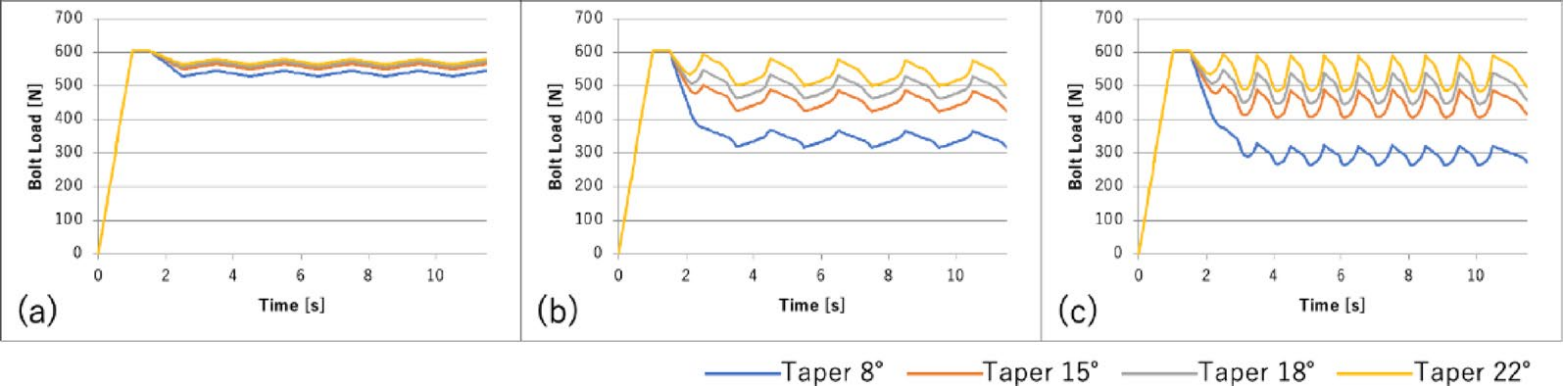
Bolt load change over time under each loading condition: **a** vertical load; **b** horizontal load (unidirectional); **c** horizontal load (bidirectional)

**Fig. 9:**
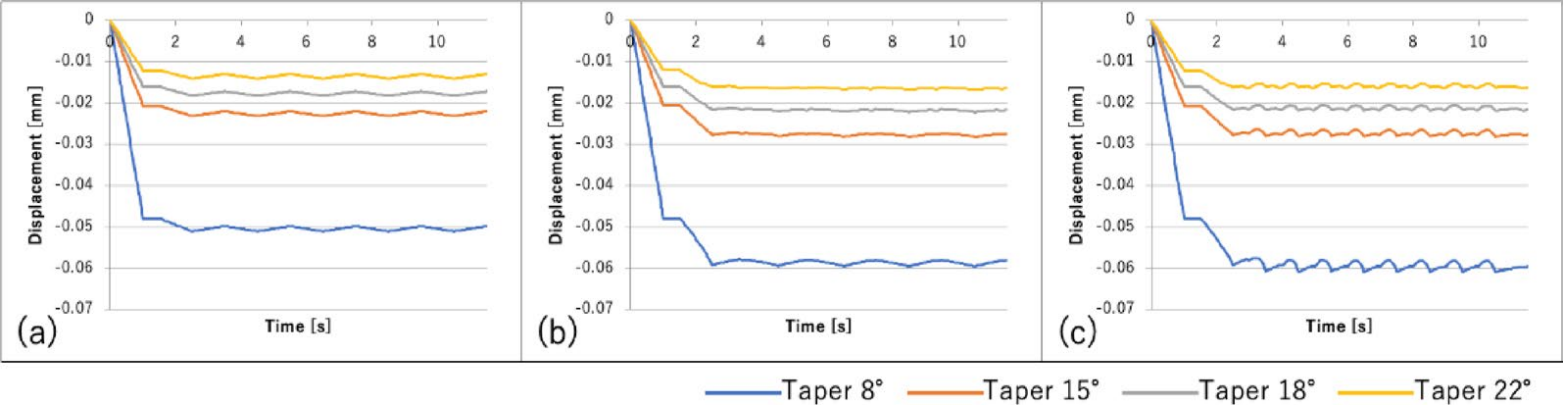
Axial abutment displacement over time under each loading condition: **a** vertical load; **b** horizontal load (unidirectional); **c** horizontal load (bidirectional)

A plot of the change in the bolt load over time is shown in Fig. 8, and a plot of the axial abutment displacement over time is shown in Fig. 9.

As the taper angle decreased, the abutment sinking and bolt load loss increased under all loading conditions.

The abutment axial displacement and bolt load loss increased in the following order: vertical < horizontal (unidirectional) < horizontal (bidirectional). For all loading conditions, the largest changes occurred in the first cycle, followed by minimal changes thereafter.

### Microgap

**Fig. 10:**
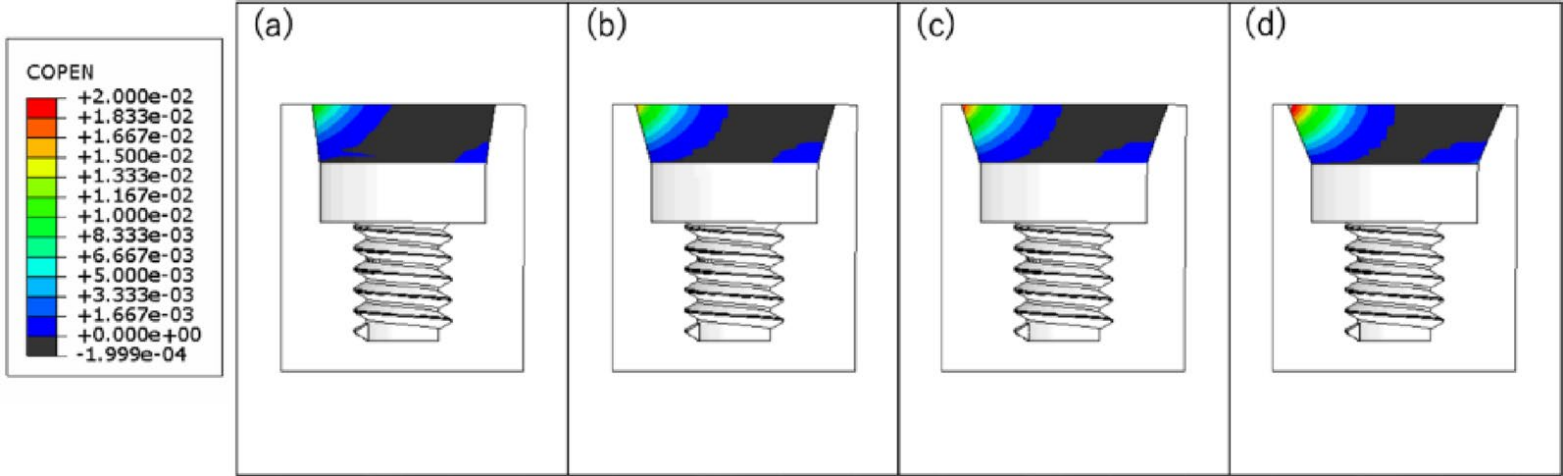
Gap between implant and abutment: **a** taper 8°; **b** taper 15°; **c** taper 18°; **d** taper 22°

**Fig. 11:**
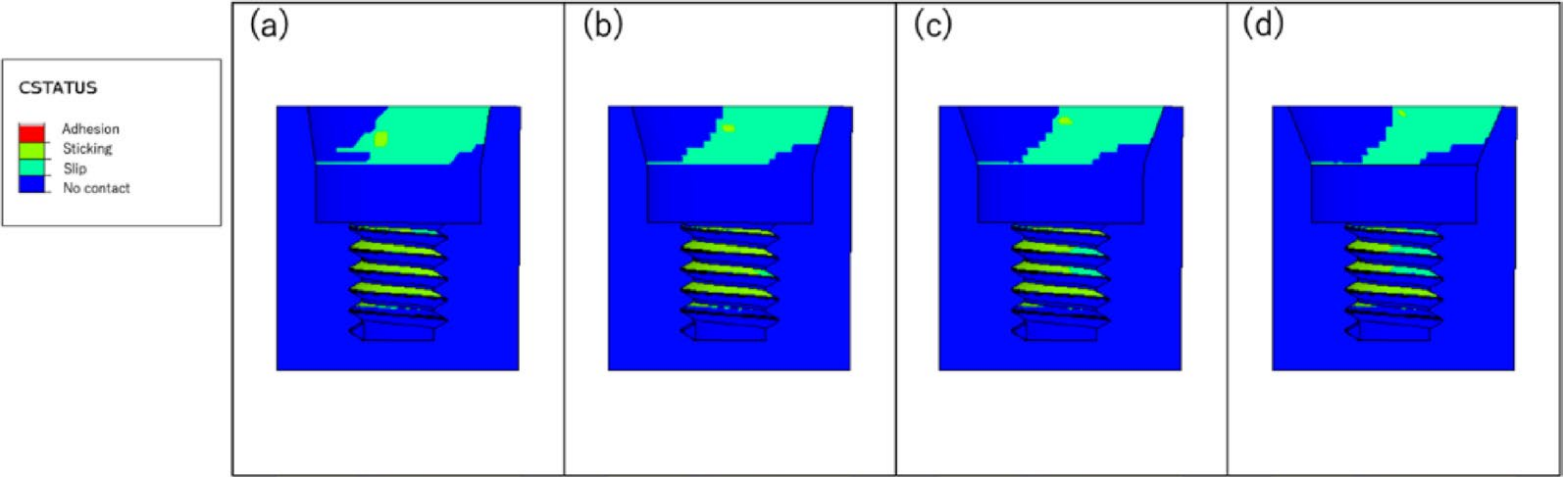
Contact between implant and abutment: **a** taper 8°; **b** taper 15°; **c** taper 18°; **d** taper 22°

Table 2 summarizes the microgaps for each angle at the first horizontal cycle, fifth unidirectional cycle, and fifth bidirectional cycle. Figure 10 shows the amount of separation between the implant body and the abutment (COPEN), and Fig. 11 shows the amount of contact when the first horizontal load was applied.

**Table 2:** Fifth unidirectional cycle, and fifth bidirectional cycle. Figure 10 shows the amount of separation between the implant body and the abutment (COPEN)

		C0PEN[mm] horizontal		
		Horizontal load 1st cycle	Horizontal load cycle (unidirectional) 5th cycle	Horizontal load cycle (bidirectional) 5th cycle
Taper angle	8°	0.0115938	0.0114111	0.0110682
	15°	0.0163997	0.0160211	0.0162394
	18°	0.0187291	0.0183062	0.0186813
	22°	0.0218536	0.0214774	0.0219129

The microgaps decreased with smaller taper angles, and also decreased across cycles, with the smallest microgaps observed after five bidirectional cycles.

At all angles, minimal line contact was observed at the bottom of the connection between the implant body and the abutment.

## Discussion

### Taper angle determination

The taper angle used in this experiment was determined by calculating the angle at which the abutment fits into the implant body as shown below.

If the coefficient of friction is μ, then, when an object slides in the slope direction, the following relationships hold:

mgsin*θ* >*µ* mgcos*θ*

*µ* < sin*θ*/cos*θ* = tan*θ*

When *µ* = 0.3, 0.3 = tan*θ*

*θ* ≒ 16.7°

Accordingly, the abutment retains press-fit (Press-fit is the condition in which the abutment remains frictionally engaged within the implant body due to normal contact pressure at the taper interface, even after the tightening preload (bolt load) is removed.) when the taper angle is smaller than 16.7°, but loses it when the taper angle is larger than 16.7°.

In this study, the following angles were compared: 8°, which is used by various implant manufacturers; 15°, which is slightly smaller than the limit of fit; 18°, which is slightly larger than the limit of fit; and 22°, which does not fit at all.

The experimental results confirm that, with taper angles of 8° and 15°, the abutment maintained press-fit even after the bolt load was released, whereas the abutment lost press-fit with taper angles of 18° and 22°. With a friction coefficient of 0.3, which was the analysis condition in this study, the friction angle was approximately 16.7°, confirming the validity of the results.

However, this friction-based threshold represents an idealized boundary. In practice, surface roughness, manufacturing tolerances, and torque variability may shift the actual transition angle. Therefore, the calculated value should be interpreted as an approximate theoretical reference rather than an absolute clinical threshold.

### Mechanistic interpretation

**Fig. 12:**
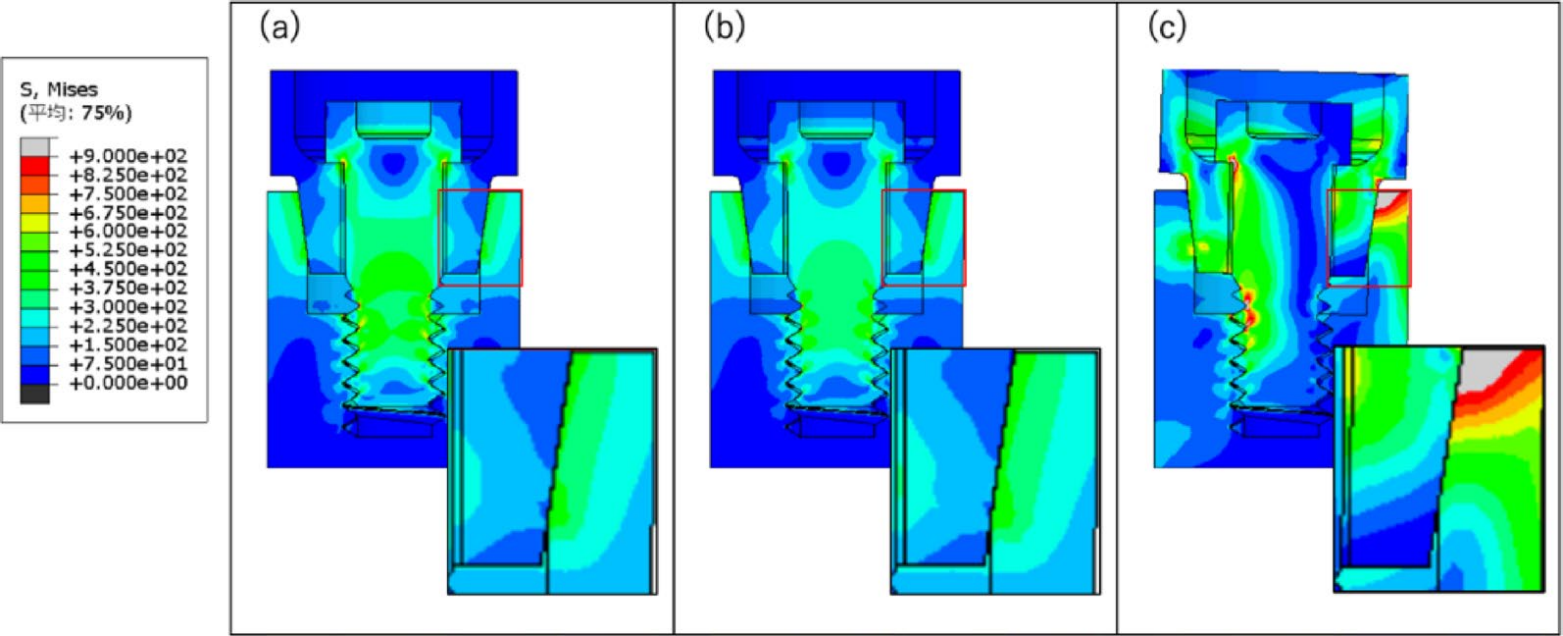
Stress distribution diagram of taper 8°: **a** bolt load; **b** vertical load; **c** horizontal load

In this study, as the taper angle decreased, the abutment axial displacement, implant stress, and bolt load loss increased, while the microgap decreased. This indicates that smaller taper angles generate higher normal contact pressure, as shown in Fig. 6, enhancing frictional wedging and reducing the interface opening (microgap). As the taper angle becomes smaller and the number of loading cycles increases, the contact pressure between the implant body and the abutment intensifies, resulting in stronger frictional engagement. Consequently, the abutment is pressed more firmly into the implant body, leading to further reduction of the microgap and improved sealing of the connection. Additionally, the abutment axial displacement and bolt load loss increased in the order of bolt load, vertical load, and horizontal load. Under bolt loading, the force acts solely in the tightening (axial) direction, producing a uniform compressive contact at the implant–abutment interface. As a result, structural deformation is minimal, and the amount of settlement remains the smallest among all loading conditions. Under vertical loading, additional axial compressive forces are applied to the top of the abutment, generating further compressive deformation within the implant–abutment assembly. Consequently, the settlement becomes greater than that observed during bolt loading alone. Horizontal loading produced the greatest effect. When lateral forces were applied, bending moments and shear stresses developed at the interface, causing partial separation of the contact surfaces. During this process, the abutment underwent slight rotational and sliding movements, which reduced the effectiveness of frictional retention. This mechanism likely explains why horizontal loading resulted in the largest increases in both abutment settlement and screw loosening (Fig. 12).

### Design and clinical implications

The findings of this study indicate a design trade‑off: smaller taper angles improve sealing (reducing microgaps) but increase stresses and the risk for screw loosening, particularly under lateral loading. Taper joint systems with small angles should ensure sufficient wall thickness, adequate axial contact length, and the use of longer abutment screws. [16, 17]. Because the taper angle was changed based on the lowest point of the joint between the implant body and the abutment, the side wall of the implant body became thicker as the taper angle became smaller. This suggests that the taper angle rather than the thickness of the implant body has a greater effect on abutment displacement and screw loosening.

Clinically, because most settlement occurs within the first lateral cycles, it is prudent to (i) verify and, if necessary, re‑torque shortly after delivery or occlusal adjustment, and (ii) perform careful lateral occlusal adjustment to minimize early settlement.

Microgaps have been reported to cause peri-implantitis [18, 19]. For patients at high risk of periodontal disease, implants with a smaller taper angle, which are thought to result in smaller microgaps, are recommended. Although smaller taper angles may contribute to reduced microgap formation, clinical outcomes such as peri-implant health are influenced by multiple biological and mechanical factors. Therefore, taper-angle selection should be interpreted as one aspect of a multifactorial clinical decision rather than a sole determinant of peri-implant disease risk.

### Limitations and future work

The FEM is affected by factors such as the mesh division of the analysis target, boundary condition and load settings, calculation time, and occurrence of singularities; therefore, it cannot fully reproduce real-world behavior. In addition, all components were modeled as linearly elastic, which may overpredict stress continuity and underrepresent local plastic deformation, particularly around the threads and contact interfaces. Incorporating elastoplastic material models in future analyses may provide more accurate stress thresholds.

In future work, mechanical testing of actual implant–abutment assemblies under comparable loading and tightening conditions will be performed to validate the FEM predictions and confirm the mechanical behavior observed in this study to improve the reliability and accuracy of the data.

## Conclusion

The findings of this study highlight the influences of the taper angle on abutment axial displacement, screw loosening, stress distribution, and microgap formation.

- When the taper angle is below a certain threshold, the abutment fits more firmly into the implant body; however, once this angle is exceeded, proper fitting is no longer possible.
- As the taper angle decreases, abutment axial displacement increases, leading to higher stress on the implant body. Horizontal loads have a greater effect than vertical loads.
- Smaller taper angles increase the likelihood of screw loosening. Additionally, the screw is more likely to loosen under horizontal loading than under vertical loading.
- Smaller taper angles reduce the microgap size.

## Data Availability

The datasets generated and/or analyzed during the current study are available from the corresponding author on reasonable request.
